# Innate immune responses in human hepatocyte-derived cell lines alter genotype 1 hepatitis E virus replication efficiencies

**DOI:** 10.1038/srep26827

**Published:** 2016-05-27

**Authors:** Pradip B. Devhare, Swapnil Desai, Kavita S. Lole

**Affiliations:** 1 Hepatitis Division, National Institute of Virology, Microbial Containment Complex, Pashan, Pune, India

## Abstract

Hepatitis E virus (HEV) is a significant health problem in developing countries causing sporadic and epidemic forms of acute viral hepatitis. Hepatitis E is a self-limiting disease; however, chronic HEV infections are being reported in immunocompromised individuals. The disease severity is more during pregnancy with high mortality (20–25%), especially in third trimester. Early cellular responses after HEV infection are not completely understood. We analyzed innate immune responses associated with genotype-I HEV replication in human hepatoma cell lines (Huh7, Huh7.5 and HepG2/C3A) using HEV replicon system. These cells supported HEV replication with different efficiencies due to the cell type specific innate immune responses. HepG2/C3A cells were less supportive to HEV replication as compared to Huh7.5 and S10-3 cells. Reconstitution of the defective RIG-I and TLR3 signaling in Huh7.5 cells enabled them to induce higher level antiviral responses and restrict HEV replication, suggesting the involvement of both RIG-I and TLR3 in sensing HEV RNA and downstream activation of interferon regulatory factor 3 (IRF3) to generate antiviral responses. Inhibition of IRF3 mediated downstream responses in HepG2/C3A cells by pharmacological inhibitor BX795 significantly improved HEV replication efficiency implying the importance of this study in establishing a better cell culture system for future HEV studies.

Hepatitis E virus (HEV) is a single-stranded positive-sense RNA virus classified in the genus *Orthohepevirus* of the family *Hepeviridae*^1^. The genome is ~7.2 kb long with short 5′-and 3′-noncoding regions (NCRs), 5′-methylguanine cap, 3′-poly (A) stretch and three open reading frames (ORF1, ORF2 and ORF3)^2^,^3^. ORF1 is translated from genomic RNA and encodes non-structural polyprotein with major domains such as methyltransferase, protease, helicase and RNA-dependent RNA polymerase that are required for virus replication^4^,^5^. ORF2 encodes capsid protein, while ORF3 encodes a small accessory protein. Both ORF2 and ORF3 are translated from a single bicistronic (subgenomic) mRNA^6^. HEV is the causative agent of hepatitis E, an acute and self-limiting disease caused by enteric transmission of the virus. Severe manifestation of hepatitis E is more common in pregnant women with high mortality rates (20–25%)^7^. Persistent HEV infections have been documented in immunosuppressed individuals^8^.

Cellular antiviral response is mediated by interferon (IFN) system and interferon induced antiviral effector genes known as interferon stimulated genes (ISGs). Two major pathways involved in sensing viral infections are Toll Like Receptor (TLR) dependent pathways and the cytosolic Retinoic acid-Inducible Gene I (RIG-I) like receptor (RLR) dependent pathways. TLRs sense pathogen components on cells surface and endosomal compartments, while, RLRs survey cytoplasm for the presence of viral double-stranded RNA (a replication intermediate) and 5′-triphosphate group containing single stranded RNA molecules^9^,^10^,^11^,^12^. Type I IFNs initiate expression of numerous ISGs, in autocrine or paracrine manner to induce antiviral state in infected cells and neighbouring cells^10^.

Molecular mechanisms associated with HEV replication and cellular antiviral responses against HEV are still not completely understood. We have previously shown that HEV can elicit inflammatory responses in human lung adenocarcinoma (A549) cells via TLR adaptors, TRIF and MyD88 and activate interferon regulatory factor 3 (IRF3) and NF-κB^13^. Dong *et al.*^14^ have demonstrated that HEV has ability to inhibit IFN-α signaling and replicate in presence of IFN-α, in A549 cell culture system. HEV mediated interferon antagonism has been shown to be mediated by macro- and papain like cysteine protease- domains of ORF1 polyprotein^15^. However, it is not yet known how liver cells detect HEV infection to initiate antiviral responses. The major obstacle has been lack of efficient liver cell culture system. Several groups have used HEV replicons and human hepatoma cell lines such as HepG2, Huh7 and PLC/PRF5 to shed some light on these aspects of HEV biology^16^,^17^,^18^.

In the present study, we used HEV replicon based cell culture system to analyse HEV induced antiviral responses. We used three hepatoma cell lines, HepG2/C3A, Huh7.5 and S10-3, which were supporting HEV replication, albeit with differential efficiencies. There was a direct correlation between induction levels of different interferon stimulated genes and HEV replication efficiencies in these cells. RIG-I and TLR3 were found to be major sensing molecules for HEV.

## Materials and Methods

### Cells

HepG2/C3A cell line was purchased from American Type Culture Collection (ATCC), S10-3 cells (a subclone of human hepatoma Huh7 cells) were a kind gift from Dr. S. Emerson (NIH, USA) and Huh7.5 cell line was from Apath LLC (Brooklyn, New York). HepG2/C3A cells were grown in Dulbecco’s modified Eagle’s medium and minimal essential medium (MEM) (mixed in 1:1 proportion) with 10% heat inactivated fetal bovine serum (FBS), 100 U penicillin/ml, and 0.1 mg streptomycin/ml while S10-3 and Huh7.5 cells were grown in Dulbecco’s modified Eagle’s medium containing 10% heat inactivated FBS, 100 U penicillin/ml, and 0.1 mg streptomycin/ml. Stocks were maintained at 37 °C while transfected cultures were incubated at 34.5 °C.

### Plasmids

HEV Rluc replicon encoding *Renilla* luciferase (Rluc) gene was a kind gift from Dr. X. J. Meng (Virginia Tech, Blacksburg, USA). This subgenomic clone has been developed from pSKHEV-2 (genotype 1 HEV infectious cDNA clone, GenBank accession No. AF444002) (19). Using HEV-Rluc replicon as template, the mutant HEV Rluc GAA was constructed (by changing conserved RdRp GDD motif to GAA) with QuickChange XL site-directed mutagenesis kit (Stratagene, La Jolla, CA). This change is known to completely stop HEV replication^18^,^19^,^20^. Plasmids bearing human RIG-I and TLR3 gene, pUNO1-hRIG-I, pUNO-hTLR3, pZERO-TLR3 (TLR3-ΔTIR; a TIR-less form of TLR3 gene) and Poly (I:C) (HMW)/Lyovec were from InvivoGen, USA.

### Generation of capped RNA transcripts and cell transfection

HEV Rluc replicon plasmid was linearized by utilizing unique Bgl II site located immediately downstream of the poly (A) tract of the HEV sequence and capped RNA transcripts were synthesized by *in vitro* transcription using mMessage mMachine T7 ultra kit (Ambion). Following transcription, DNA template was removed by DNase I treatment, transcribed RNA was purified by lithium chloride precipitation method as per the manufacturer’s instructions and quantified on Nanodrop spectrophotometer (ND-1000, Nanodrop technologies). Integrity of the transcripts was checked by doing denaturing agarose gel electrophoresis. For each experiment, cells were grown up to 60–70% confluence in 24-well cell culture plates and washed with serum free medium, OptiMEM (Invitrogen, Life technologies) prior to transfection. Cells were transfected with capped RNA transcripts, diluted appropriately in OptiMEM (2 μg/well of the 24 well plate) using 1,2-dimyristyl Rosenthal inhibitor ether (DMRIE-C) reagent (Invitrogen) as per the manufacturer’s instructions. Cells were co-transfected with Firefly luciferase plasmid DNA (pGL-3 promoter vector, 100 ng/well) along with HEV-Rluc RNA to normalize cell transfection efficiency and *Renilla* luciferase signals. For gene expression analysis, transfections were carried out similarly without including firefly luciferase plasmid DNA. After 4 h of incubation at 34.5 °C, transfection mixture was replaced with DMEM containing 10%FBS. All cell transfections were carried out in triplicates and each set of experiments was repeated twice/thrice. For plasmids, cell transfections were carried out with Lipofectamine 2000 transfection reagent (invitrogen) as per the manufacturer’s instructions.

### Reporter gene assay

Monolayer of the cells transfected with RNA was washed two times with phosphate buffered saline, cells were lysed in 100 μl of 1X Passive Lysis Buffer (Promega) and lysates were immediately frozen at −80 °C until use. For the assay, samples were thawed, centrifuged at 10,000 RPM for 2 min and 20 μl cell lysates were used for measuring dual luciferase activities (*Renilla* luciferase: Rluc and firefly luciferase: FLuc) using Dual luciferase assay system (Promega) and readings were taken on the Perkin Elmer 2030 Reader (Victor X3). Rluc values were normalized with FLuc values at respective time points.

### Treatment of the cells with IFN-α and BX795 inhibitor

Before transfection with RNA, cells were pre-treated for 2 h with 1 μM BX795 (InvivoGen) while IFN-α (500–1,000 U/ml) (Sigma) was added to the culture medium after 4 h of cell transfection with RNA. Cell treatment with BX795 or IFN-α was continued after transfection till the end point of the respective experiment. Cells remained untreated during the 4 h transfection period.

### Gene Expression profiling by TaqMan Low Density Array (TLDA)

Antiviral pathway genes (n = 95) and 18 s rRNA as endogenous control were chosen and the array cards were procured from Applied Biosystems (USA). Gene expression profiling was carried out as described previously^13^.

### Quantitative real-time PCR (qRT-PCR)

Individual SYBR green-based quantitative reverse transcription PCR assays were performed for selective genes. The cDNAs prepared as described previously^13^ were analyzed on 7300 Real-Time PCR system (Applied Biosystems, USA). GAPDH was used as a housekeeping gene to normalize the RNA input. RNA from mock transfected cells was used as the calibrator and relative gene expression analysis was carried out using SDS2.2 software (Applied Biosystems, USA).

### Immunoblotting

Immunoblotting was carried out as described previously^13^. The primary antibodies used were anti-RIG-I (IMGENEX), mAb anti-phospho IRF3 (Ser396), anti-TLR3 (Cell Signaling Technology, Beverly, MA), anti-IRF3 and anti-actin (Sigma).

## Results

### Differential replication efficiencies of HEV in different hepatoma cell lines

**Figure 1:**
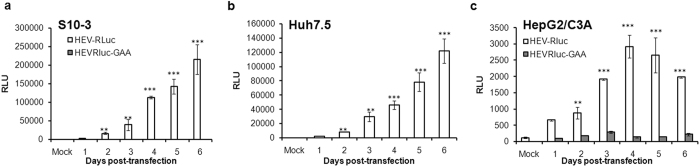
Differential replication efficiencies of HEV in different human hepatocyte derived cell lines. S10-3 (**a**), Huh7.5 (**b**) and HepG2/C3A (**c**) cells were transfected with either wild-type HEV Rluc or HEV Rluc GAA (GDD mutant) RNA (2 μg/well). Cells were co-transfected with firefly luciferase plasmid DNA (pGL-3 promoter vector, 100 ng/well) to normalize cell transfection efficiency and the *Renilla* luciferase signal. Cell associated *Renilla* luciferase activity was checked to monitor HEV replication from 1-6 days. The data represents mean ± SD of three independent triplicate set of experiments, [** and ***represent p < 0.01 and p < 0.001 respectively, statistical comparisons were done by one-way analysis of variance (ANOVA) between mock transfected cells, and cells transfected with HEV-Rluc RNA].

Human hepatoma cell lines HepG2/C3A and Huh7 derived clonal cell lines S10-3 and Huh7.5 were transfected with capped RNA transcripts generated from HEV Rluc (original wild type) and GDD mutant (HEV Rluc GAA) replicons and processed to measure luciferase activities which would reflect viral RNA replication. HEV Rluc is subgenomic replicon which expresses Rluc in place of ORF2 (capsid) protein. Detection of Rluc activity means successful negative strand RNA intermediate synthesis, followed by positive strand genomic and subgenomic RNA synthesis. As seen from the Rluc activities, all three hepatoma cell lines were supporting HEV RNA replication, however, replication efficiencies differed. HepG2/C3A cells were found to be less supportive as compared to S10-3 (p = 0.0057, for the 6^th^ day) and Huh7.5 (p = 0.0026, for the 6^th^ day) cells. *Renilla* luciferase activity of HEV Rluc GAA remained negligible till 6 days without any increase from the base level values seen after 24 h in Huh7 derived cell lines S10-3, Huh7.5 (data not shown) and HepG2/C3A cells (Fig. 1a–c).

### Cell-specific antiviral responses

Since there was a significantly low level HEV replication in HepG2/C3A cells as compared to other two cell lines, we decided to analyze antiviral status of these cells. For that, TaqMan Low Density Array (TLDA)^13^ cards were used to analyze gene expression levels of different innate immune pathway genes in the transfected cells. Total RNA was isolated at 24 h and 96 h post-transfection and processed for the gene expression analysis as described previously^13^ (Table 1). Results obtained with low density arrays showed differential expression of genes which were functionally categorized as follows-.

#### Pattern Recognition Receptors (PRRs)

Transcription levels of viral RNA sensing receptors RIG-I (DDx58), Mda5 (IFIH1) and TLR3 were higher in HepG2/C3A cells transfected with wild-type HEV Rluc RNA (~2 folds higher) as compared to HEV Rluc GAA RNA at 24 h. This difference in gene expression was more noticeableat 96 h post-RNA transfection suggesting the replication specific effect. However, expression levels of these genes in S10-3 and Huh7.5 cells, transfected with both HEV Rluc and HEV Rluc GAA RNA were significantly low. This suggested that S10-3 and Huh7.5 cells were either unable to detect replicating HEV RNA or had impaired downstream antiviral pathways.

#### Interferon regulatory factors (IRFs)

Activation of IRFs is the downstream signaling component after sensing of virus associated PAMPs. The transcription factors such as IRF1, IRF3, IRF7 and IRF9 are known to play important role in initiating cellular antiviral responses^21^. HepG2/C3A cells showed increased expression levels of IRF1, IRF3, IRF7 and IRF9 genes (4.6, 2.0, 3.9 and 18.3 folds respectively) as compared to mock transfected cells at 24 h. Expression levels of IRFs reverted back to basal level at 96 h, except for IRF9 which remained induced ~6 fold compared to GAA mutant RNA. IRF9 is the component of ISGF3 complex and aids in amplification of ISG response. This clearly corroborated with ongoing viral RNA replication and amplification of innate response in HepG2/C3A cells. There was no change in the expression levels of these genes in S10-3 and Huh7.5 cells.

#### Type I Interferons (IFNs) and Interferon stimulated genes (ISGs)

There was no significant increase in the levels of transcripts for the type I IFN genes, IFN-α, IFN-β and IFN-ω in any of the hepatoma cells transfected with HEV Rluc or HEV Rluc GAA.

Interferon stimulated genes such as ISG15, ISG20, PKR, IFI27, IFIT1 (ISG56), IFIT2 (ISG54), MX1, MX2, OAS1, RSAD2/ viperin, STAT1, STAT2, STAT3 and TNFSF10/TRAIL showed significantly higher induction in HepG2/C3A cells transfected with HEV Rluc RNA as compared to HEV Rluc GAA (Table 1). Point noteworthy here was that HepG2/C3A cells showed up regulation of above genes in response to HEV GAA Rluc, as *in vitro* transcribed capped RNA transcripts are known to have some amount of uncapped RNA (with 5′ phosphate ends), which is a potential inducer of innate immune response. However, expression levels of all ISGs were significantly higher in cells transfected with HEV Rluc at 24 h and 96 h. These observations indicated that HepG2/C3A cells were sensitive in detecting both single stranded RNA and double stranded RNA intermediates. On the other hand, S10-3 and Huh7.5 cells were less sensitive.

### IFN-α inhibits HEV RNA replication in Huh7.5 cells

**Figure 2:**
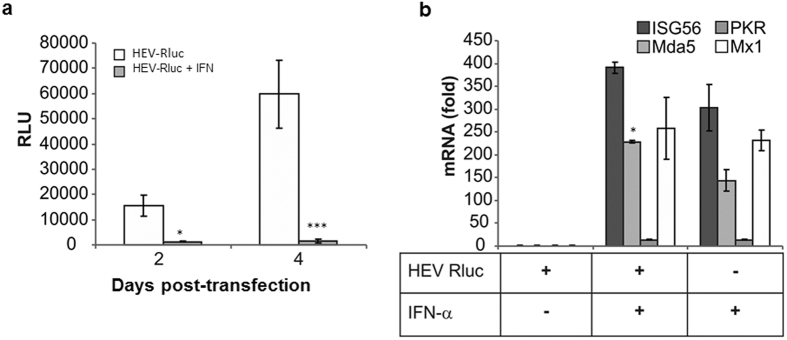
Interferon-α treatment inhibits HEV replication in Huh7.5 cells. (**a**) Huh7.5 cells transfected with HEV Rluc replicon RNA were treated with IFN-α (1,000 U/ml) 4 h post transfection. Cells were assayed for luciferase activity to monitor HEV RNA replication as described in Fig. 1, [* and ***represent p < 0.05 and p < 0.001 respectively, statistical comparisons were done by one-way analysis of variance (ANOVA) between cells transfected with HEV-Rluc RNA, and cells transfected with HEV-Rluc RNA and treated with IFN-α]. (**b**) Expression of ISGs; *ISG56, Mda5, Mx1* and *PKR* genes in Huh7.5 cells was checked by SYBR-green based qRT-PCR, 24 h post RNA transfection (mRNA levels (fold) were calculated in relation to the levels of respective genes in mock transfected cells). The data represents mean ± SD of two independent triplicate sets of experiments [*represents p < 0.05, comparison between IFN-α treated cells and cells transfected with HEV-Rluc RNA and treated with IFN-α].

To confirm direct correlation of HEV replication with ISGs, we exposed Huh7.5 cells to IFN-α (1,000 U ml^−1^) 4 h after transfection and measured luciferase activity. Cells exposed to IFN showed significant suppression of HEV replication as compared to unexposed cells (Fig. 2a). These results were in agreement with previous reports that have shown functional impairment of pathogen recognition receptor/s in these cells^22^,^23^. To confirm antiviral status of IFN exposed Huh7.5 cells, we analysed expression levels of representative ISGs by SYBR green based quantitative real time PCR assay and detected considerably higher levels of ISG56, IFIH1 (Mda5) and Mx1 in cells transfected with HEV Rluc and then treated with IFN (Fig. 2b). These results showed that downstream antiviral response pathways are intact in Huh7.5 cells since externally added IFN-α could induce ISGs and restrict HEV replication.

### Reconstitution of RIG-I and TLR3 signaling pathways in Huh7.5 cells can restrict HEV replication

**Figure 3:**
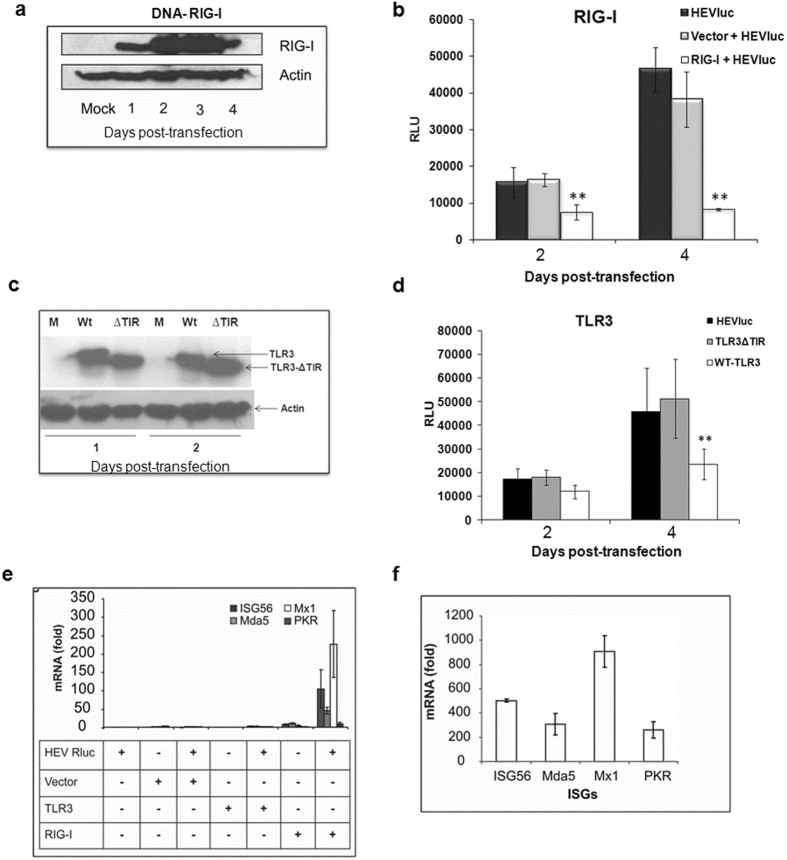
RIG-I and TLR3 are the pattern recognition receptors in sensing HEV RNA replication. (**a**) RIG-I expression in Huh7.5 cells: Cells either mock transfected or transfected with RIG-I expression plasmid (pUNO-hRIG-I) were analyzed by immunoblotting for RIG-I protein levels at 24, 48, 72 and 96 h respectively (lanes 1–4). Actin was used as a loading control. (**b**) RIG-I complementation in Huh7.5 cells inhibits HEV RNA replication: Huh7.5 cells were either mock transfected or transfected with empty vector or with RIG-I expression plasmid. After 24 hours, cells were transfected with HEV Rluc RNA and monitored by luciferase assay at 2 and 4 days post RNA transfection respectively. (**c**) TLR-3 and TLR3-ΔTIR expression in Huh7.5 cells: Cells either mock transfected or transfected with TLR3 expression plasmids (pUNO-hTLR3 and pZERO-TLR3) were analyzed by immunoblotting for TLR3 protein levels at 24 and 48 h respectively. TLR3-ΔTIR is a TIR domain-less form of the TLR3 gene showing lower molecular weight compared to wild-type TLR3 (TLR3-ΔTIR recognizes its ligands but is unable to induce the signaling). Actin was used as a loading control. (**d**) TLR3 complementation in Huh7.5 cells inhibits HEV RNA replication: Huh7.5 cells were transfected with TLR3 or TLR3-ΔTIR expression plasmids 24 h prior to transfection with HEV Rluc RNA and monitored by luciferase assay at 2 and 4 days post transfection respectively [**represents statistical comparison by one-way analysis of variance (ANOVA) with cells transfected with empty vector or TLR3-ΔTIR expression plasmid and showed p < 0.01 in figures (**b**,**d**)]. (**e**) RIG-I and TLR3 complementation restore antiviral response in Huh7.5 cells: Cells expressing RIG-I and TLR3 were transfected with HEV Rluc RNA and expression of ISGs *ISG56, MDA5, Mx1* and *PKR* were assessed by SYBR-green based qRT-PCR at 24 h post RNA transfection. (**f**) Antiviral response in HepG2/C3A cells: HepG2/C3A cells transfected with HEV Rluc RNA were analyzed for ISG expression similarly as in (**e**) with Huh7.5 cells as a control. The data represents mean ± SD of three independent triplicate sets of experiments. Cropped blots are used in the main figure and full length blots are included in Supplementary Figure 1.

Huh7.5 cell line is known to have defective RIG-I signaling due to a point mutation within its CARD-like homology domain^23^. To assess whether RIG-I has any role in detecting double stranded RNA during HEV replication, we decided to reconstitute RIG-I pathway by transfecting these cells with plasmid containing RIG-I expression cassette, confirmed the expression (Fig. 3a) and assessed HEV replication in these cells. As shown in Fig. 3b, HEV replication remained low at 2 and 4 days post HEV RNA transfection in cells expressing functional RIG-I, whereas, cells transfected with empty vector showed significantly higher HEV replication at 4 days. Thus, RIG-I complementation in Huh7.5 cells resulted in phenotypic switch from hyper permissive to a relatively non permissive phenotype characterized by suppression of viral RNA replication. These results suggested important role of RIG-I as pathogen recognition receptor in detecting HEV RNA.

Similar to RIG-I, Huh7 cells are reported to be defective in TLR3 signaling due to lower expression of TLR3^22^. By reconstituting TLR3 pathway in Huh7.5 cells (Fig. 3c) we assessed the role of TLR3 in sensing HEV RNA replication. It was observed that elevated expression of functional TLR3 in Huh7.5 cells significantly inhibited HEV replication as compared to defective TLR3 (TLR3-ΔTIR), lacking the TIR signaling domain (Fig. 3d), however this inhibition was not as pronounced as that seen with RIG-I. These results indicated that both RIG-I (in the cytoplasm) and TLR3 (in the endosomal compartments) can function as sensors in hepatocytes for HEV RNA.

To confirm antiviral status of the cells we assessed levels of representative ISGs in the RIG-I expressing cells. As expected, the levels of ISG56 (105 fold), Mda5 (47 fold), Mx1 (227 fold) and PKR (10 fold) were significantly increased only in cells transfected with plasmid containing functional RIG-I gene and not with the empty vector. Similar analyses with TLR3 complementation showed comparatively lower levels of ISGs (~2.5 to 4 folds) as compared to RIG-I complemented cells (Fig. 3e) indicating TLR3 being less effective in detecting HEV replication as compared to RIG-I. HepG2/C3A cells transfected with HEV Rluc RNA showed increased expression of ISGs in agreement with the TLDA results (Fig. 3f). Overall, these results showed involvement of RIG-I and TLR3 pathways in restricting HEV replication in hepatoma cells.

### Inhibition of pattern recognition receptor signaling enhances HEV replication

**Figure 4:**
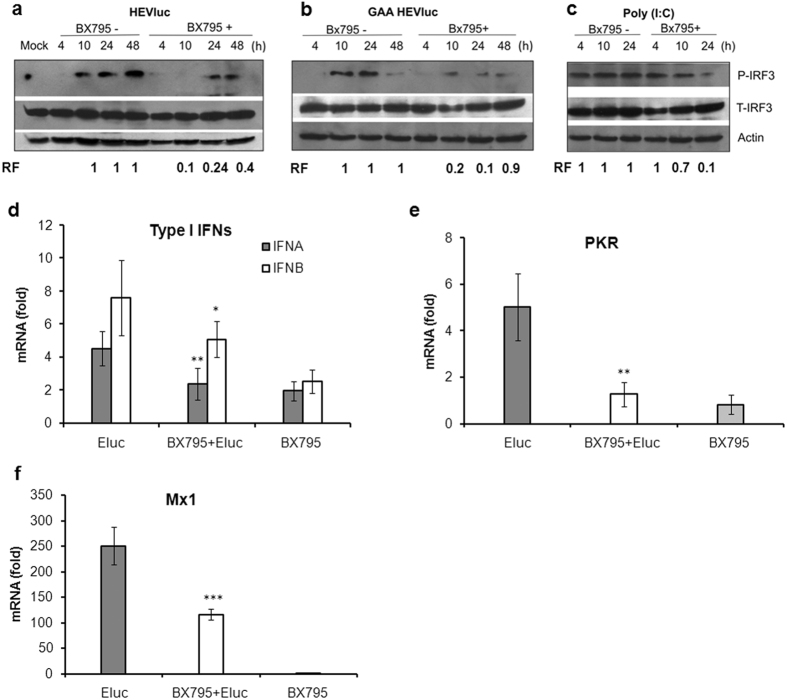
BX795 blocks the phosphorylation and activation of IRF3 leading to suppression of interferon stimulated genes (ISGs). HepG2/C3A cells, untreated or treated with BX795, were transfected with (**a**) HEV Rluc RNA, (**b**) HEV Rluc GAA and (**c**) double stranded RNA analogue (poly I:C) and processed for immunoblotting for the detection of phosphorylated IRF3 (P-IRF3, Ser396). The blot was reprobed for the detection of total IRF3 (T-IRF3) and actin at indicated time points.The relative protein band density for p-IRF3 was normalized with actin and compared with mock treated and BX795 treated cells at different time points and mentioned as relative fold (RF) values. Cropped blots are used in the main figure and full length blots are included in Supplementary Figure 1. (**d**–**f**) HepG2/C3A cells left untreated or treated with BX795 were transfected with HEV Rluc RNA and processed for qRT-PCR of type I IFN genes (*IFNA* and *IFNB*) and ISGs (*PKR and Mx1*) at 24 h post transfection. The data represents mean ± SD of three independent experiments [*, ** and ***represent p < 0.05, p < 0.01 and p < 0.001 respectively, statistical comparisons were done by one-way analysis of variance (ANOVA) between HEV Rluc and BX795 + HEV Rluc].

After confirming the role of RIG-I and TLR3 in sensing HEV RNA, we checked the effect of inhibition of IKK related kinase, TBK1 (TANK-binding kinase 1) and IKKε complex, which acts as the common mediator in downstream signaling for both RLRs (RIG-I/Mda5) and TLR3. HepG2/C3A cells were exposed to BX795, a cell permeable inhibitor which is relatively specific for TBK1/IKKε, known to block phosphorylation, nuclear translocation and transcription activity of IRF3^24^. To analyse possible changes in the IRF3 phosphorylation status of BX795 treated cells, cells were transfected with either double stranded RNA analogue, poly (I:C) or HEV Rluc. There was a significant reduction in the levels of IRF3 phosphorylation in BX795 treated cells as compared to untreated cells (Fig. 4a,b). Importantly, there were comparable levels of IRF3 phosphorylation at the early time points after transfection with HEV Rluc or HEV Rluc GAA (at 10 and 24 h) in BX795 untreated cells, which was also evident from the elevated expression levels of antiviral genes. However, there was a selective increase in IRF3 phosphorylation at the later stages (48 h) only in cells transfected with HEV Rluc, indicating requirement of ongoing viral RNA replication (double stranded RNA formation). BX795 treatment also inhibited poly (I:C) triggered IRF3 phosphorylation at 10 and 24 h (Fig. 4c). Inhibition of IRF3 phosphorylation was also evident from the down regulation of transcripts for type I IFNs and ISGs such as PKR and Mx1 in BX795 treated cells as compared to untreated cells (Fig. 4d–f).

**Figure 5:**
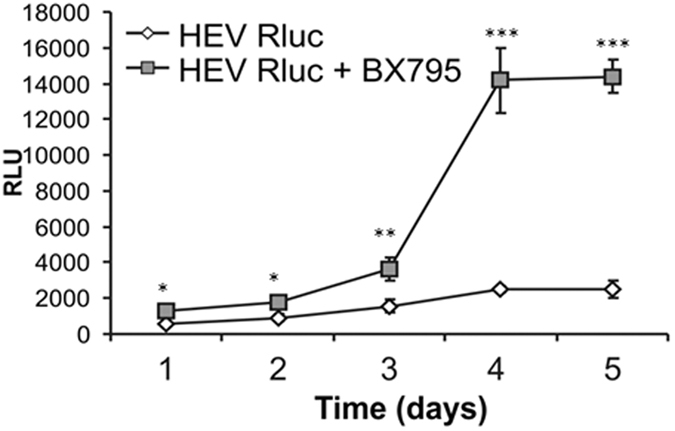
Inhibition of IFN signaling enhances HEV replication efficiency. HepG2/C3A cells untreated or treated with BX795 were transfected with HEV Rluc RNA and viral RNA replication was monitored by luciferase assay from 1–5 days post transfection. The data represents mean ± SD of three independent triplicate sets of experiments [*, ** and ***represent p < 0.05, p < 0.01 and p < 0.001 respectively, statistical comparisons were done by one-way analysis of variance (ANOVA) between untreated and BX795 treated cells at indicated time points].

Treatment of HepG2/C3A cells with 1 μM BX795 showed significant improvement in HEV replication. On monitoring of the cells that were transfected with HEV Rluc further, a steady increase in the replication was observed from the days 1–5 in BX795 treated cells as compared to untreated cells (Fig. 5). Overall, these results confirmed the role of IRF3 mediated antiviral pathways in restricting HEV RNA replication in HepG2/C3A cells.

## Discussion

Though there are reports on efficient cell culture models for genotype 3 and 4 viruses^25^, better cell culture systems are still lacking for genotype 1 HEV studies. We used HEV replicons and human hepatoma cells such as HepG2/C3A, Huh7.5 and S10-3 for HEV replication analysis and observed that these cells do not support HEV replication at comparable levels. In the present study, we addressed this issue by utilizing luciferase reporter based subgenomic HEV replicon (HEV Rluc) that allowed quantitation of HEV replication in terms of *Renilla* luciferase activity. Real-time PCR based assay could not be used for the quantitation due to two reasons, high background of replicon RNA used for the transfections and very low replication efficiency of the replicon. The quantitative luciferase assay showed that HEV replicates more efficiently in Huh-7 derived cell lines, S10-3 and Huh7.5, as compared to HepG2/C3A cells (Fig. 1). Similar observations have been reported by Emerson *et al.*^17^. Cellular antiviral responses were the most obvious cause for the restricted virus replication. It is also known that Huh7 cells have impaired TLR3 pathway while, Huh7.5 are defective in both RIG-I and TLR3 pathways. Hence, it was understood that these cells were able to restrict HEV replication less efficiently as compared to HepG2/C3A cells.

Our gene expression analysis experiments confirmed that there were comparatively higher levels of the pattern recognition receptor (PRRs) and type I IFN pathway gene transcripts in HepG2/C3A cells as compared to S10-3 and Huh7.5 cells (Table 1). Though expression levels were higher in HepG2/C3A cells transfected with HEV Rluc as well as HEV Rluc GAA (mutant) in comparison with mock transfected cells, levels were further elevated in cells transfected with HEV Rluc. This indicated that cells could sense secondary structures in RNA immediately upon transfection and triggered antiviral responses and upon sensing double stranded RNA replication intermediates enhanced this induction further. To confirm involvement of type I IFNs in eliciting antiviral environment, we exposed Huh7.5 to IFN-α, and detected significant inhibition of HEV replication, due to induction of ISGs (Fig. 2). This confirmed presence of intact downstream pathway/s in these cells. Huh7 cells have proven to be nearly unique in their ability to support autonomous replication of hepatitis C virus^26^ and hepatitis A virus^27^ RNA replicons.

Pattern recognition receptors (PRR) recognizing HEV associated molecular patterns are still unknown. Considering presence of defective RIG-I/TLR3 signaling and HEV permissiveness of Huh7.5 cells, we used these cells further to identify HEV PRRs. We reconstituted RIG-I and TLR3 signaling by expressing functional proteins exogenously in Huh7.5 cells and observed that both viral RNA sensing pathways are involved in recognizing HEV RNA. It was seen that RIG-I acted as an immediate sensor of HEV RNA (at 2 days) while, TLR3 sensed replicative intermediates comparatively later (at 4 days) (Fig. 3b,d). This was expected since, ssRNA with 5′-phosphate groups and dsRNA are both potential RIG-I ligands^12^, while TLR3 senses only dsRNA. Reconstitution of the RIG-I signaling in Huh7.5 cells resulted in comparatively higher levels of ISGs that resulted in efficient virus inhibition as compared to TLR3 reconstitution (Fig. 3e). The difference could be also due to specific cellular locations of these RNA sensors, as TLR3 mainly resides in endosomal compartments while RLRs remain in cytoplasm. Some RNA viruses release their viral RNA genomes into cytoplasm allowing cytosolic PRRs to be activated^12^. However, activation via endosomal TLRs remains limited, since most of the viral RNA remains membrane-associated in viral replication complexes, and probably safeguarded from the TLR3 surveillance. Proposed site of HEV replication is assumed to be endoplasmic reticulum^28^ and hence less accessible to TLR3.

Following infection, latent IRF3 in cytoplasm is known to get translocated into nucleus upon TBK1/IKKε mediated phosphorylation and result into transcription of IFN and IFN stimulated genes^29^. Both TLR3 and RIG-I pathways are known to converge into downstream IRF3 activation. Since both RIG-I and TLR3 were detected as HEV RNA sensors, we decided to inhibit downstream IRF3 activation. When HepG2/C3A cells were exposed to BX795, an inhibitor of TBK1/IKKε^24^, there was a significant decrease in IRF3 phosphorylation, low level induction of ISGs and these resulted finally in improving HEV replication. Similar enhancement in lentiviral transduction efficiency has been shown in BX795 treated human and mouse cell lines^30^.

Various human hepatoma cell lines HepG2, PLC/PRF5 and Huh7 and non-hepatoma cell lines Caco2 (human colon carcinoma) and A549 are known to support HEV replication (13, 17, 25, 31–33). However, none of these cell culture systems can provide high titers of infectious virus in culture supernatants. Amongst these cells, Huh7 derived cell lines are comparatively better, however, HEV infection needs to be initiated by transfecting these cells with replicon RNA instead of direct infection with native virus particles. Although PLC/PRF5 cell line was reported to propagate genotype 3 HEV^32^,^33^, this cell line contains hepatitis virus B surface antigen (HBsAg) and thus cannot be used to study HEV specific innate immune response. On the other hand, HepG2/C3A cells can be directly infected with virus particles but they restrict virus replication. HepG2/C3A cells are thought to have higher density of HEV receptors on their surface as compared to Huh7 derived cell lines^31^ and hence are a better system to assess virus infectivity. With present observations, we feel BX795 treated HepG2/C3A cells could be used as a cell culture model for genotype 1 HEV studies.

In conclusion, we observed direct correlation between HEV replication and levels of antiviral responses in different hepatoma cell lines. RIG-I and TLR3 pattern recognition receptors were mainly responsible for HEV RNA detection in these cells. Inhibition of IRF3 activation by BX795 in HepG2/C3A cells prevented synthesis of interferon stimulated genes and improved HEV replication in these cells. BX795 could be used to improve HEV replication in HepG2/C3A cells for its use as a model system to study HEV biology.

## Additional Information

**How to cite this article**: Devhare, P. B. *et al.* Innate immune responses in human hepatocyte-derived cell lines alter genotype 1 hepatitis E virus replication efficiencies. *Sci. Rep.* **6**, 26827; doi: 10.1038/srep26827 (2016).

## Supplementary Material

Supplementary Information

## Figures and Tables

**Table 1:** Gene expression profiling in Hepatoma cell lines transfected with HEV Rluc and HEV Rluc GAA RNA.

Pathway/ Function	Gene Name	RQ Values (Fold change) determined by TLDA											
		HepG2/C3A				S10-3				Huh7.5			
		24 h		96 h		24 h		96 h		24 h		96 h	
		HEV Rluc	GAA	HEV Rluc	GAA	HEV Rluc	GAA	HEV Rluc	GAA	HEV Rluc	GAA	HEV Rluc	GAA
PRRs	DDx58	32	15.2	11.8	4.2	4.4	1.1	0.7	0.9	1.5	1.3	0.9	0.8
	IFIH1	142	80	7.2	2.6	5.8	1.5	0.8	1.2	2.1	1.5	1.2	1.2
	TLR3	157	124	19.7	0.6	3.5	0.7	2.2	1.3	3.9	1.8	2.2	1.5
Interferons	IFNA1	3	1.5	7.5	0.5	3.2	0.7	1.2	0.7	3.8	1.4	3.7	0.3
	IFNB1	3.3	2	5.7	0.6	2.6	0.7	1	0.8	2.2	1.7	2.1	0.4
	IFNW1	3.5	2	8.1	0.5	3	0.7	1.2	0.8	3.4	2.6	2.7	0.4
Interferon regulatory factors (IRFs)	IRF1	4.6	2.7	1.4	0.4	2.1	0.6	0.9	0.4	1.7	1.8	1	1
	IRF3	2	1.2	0.99	0.2	1.3	0.7	0.4	0.3	1.2	1.4	0.7	0.6
	IRF7	3.9	2.6	1.1	0.1	3.2	1.1	1.9	0.6	ND	ND	ND	ND
	IRF9	18.3	9.8	6.9	1.2	9.2	2.7	1.3	1.6	1.3	1.1	0.6	1.3
Interferon stimulated genes (ISGs)	ISG15	108	80.2	6.8	1.7	6	4	1.6	1.37	1.3	1.2	0.7	1
	ISG20	24.3	16.5	2	0.5	2.8	1.4	2.9	1	1.4	1.4	0.9	1
	EIF2AK2/PKR	10.2	6.3	3.7	1.3	4.3	1.1	0.7	0.6	1.5	1.1	1	0.7
	GBP1	78.3	52	1.2	1.3	7.4	1.7	3.2	1.6	2.3	2.1	5.1	1
	IFI27	901	600	140.7	63.4	6.7	2.5	3.5	1.3	1	0.7	1.9	0.7
	IFIT1/ISG56	582	344.7	5.6	1.4	28.6	9.5	7.1	2	1.3	0.9	5.9	0.73
	IFIT2/ISG54	13990	7424	120	65.2	15.9	3.6	1.4	1.5	2.9	3.1	2.9	1
	MX1	865.5	372	98	39.6	50.4	14.7	9.3	2.7	1.1	0.9	7.3	0.6
	MX2	16.6	10.2	1.8	0.9	1.3	0.4	0.2	0.3	1	1	0.3	0.6
	OAS1	446	315	27.5	9.6	15.5	5.7	4.5	1.4	1.2	1	1.4	1.3
	RSAD2/Viperin	6873	4987	23.5	0.9	4.6	1.3	0.9	0.7	ND	ND	ND	0.9
	SOCS1	4.5	2.4	3.8	1.2	1.2	0.6	0.5	0.3	1.3	1.1	1	0.4
	STAT1	25	13.3	1.3	2.7	2	0.8	0.6	0.6	1.1	1.4	0.8	0.7
	STAT2	4.5	2.2	1.2	0.7	1	0.4	0.2	0.4	1.3	1.3	0.6	0.5
	STAT3	1.2	0.7	2.8	1.5	2.7	0.7	0.3	0.8	2	1.5	1.1	0.9
	B2M	7.3	5.2	4.9	0.8	1.9	0.7	0.6	0.7	0.9	1	0.9	0.6
	TNFSF10/TRAIL	34	20	2.5	0.8	2	0.4	0.9	0.3	1.2	0.8	1	0.9

Gene expression results are given as averages from duplicate cultures and expressed as fold change (RQ value) compared to the mock transfected cells (Fold change ≥ 2 considered to be up-regulated genes) (ND, Not determined).
