# Supplementary material for: Innate immune responses in human hepatocyte-derived cell lines alter genotype 1 hepatitis E virus replication efficiencies
Source: Sci Rep. 2016 May 27;6:26827. doi: 10.1038/srep26827 (PMC4882509; doi:10.1038/srep26827)
Supplement: Supplementary Information [file srep26827-s1.pdf]

Supplementary figures

Title: Innate immune responses in human hepatocyte-derived cell lines alter genotype 1 hepatitis E virus replication efficiencies

Pradip B. Devhare, Swapnil Desai, Kavita S. Lole *

Hepatitis Division, National Institute of Virology, Microbial Containment Complex, Pashan, Pune, India.

***For Correspondence**

Kavita S. Lole

E-mail: lolekavita37@yahoo.com

Tel: +91-20-25871194; Fax: +91-20-25871895.

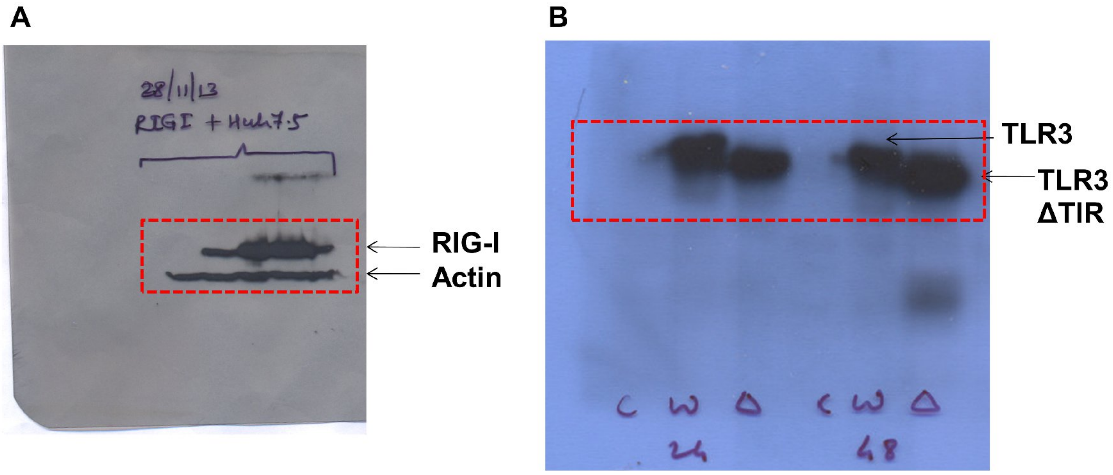
A
28/11/13
RIGI + Huh7.5
RIG-I
Actin
B
TLR3
TLR3
ΔTIR
C W Δ C W Δ
24
48

C **HEVluc**

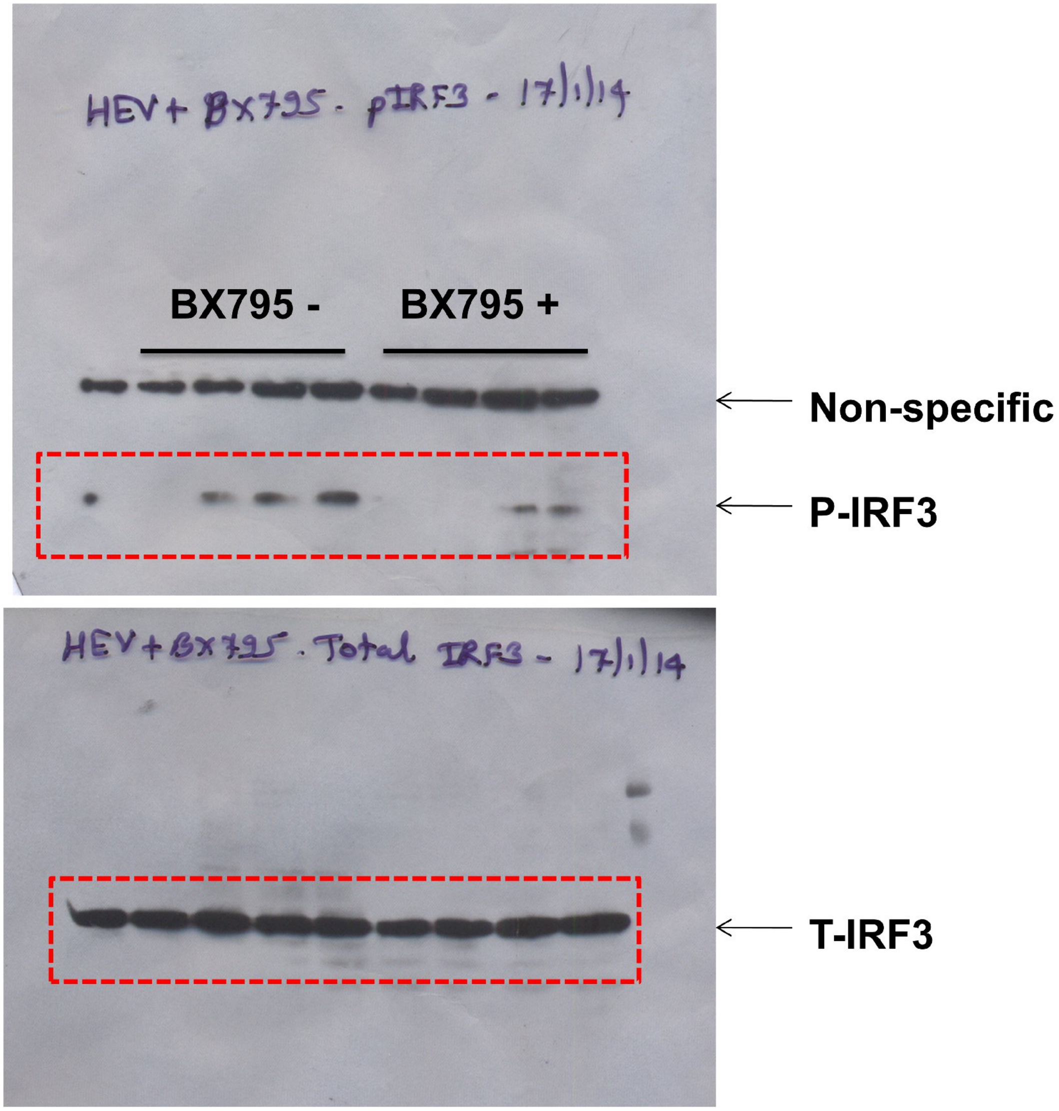
HEV + BX795 - pIRF3 - 17/1/14
BX795 -
BX795 +
Non-specific
P-IRF3
HEV + BX795 . Total IRF3 - 17/1/14
T-IRF3

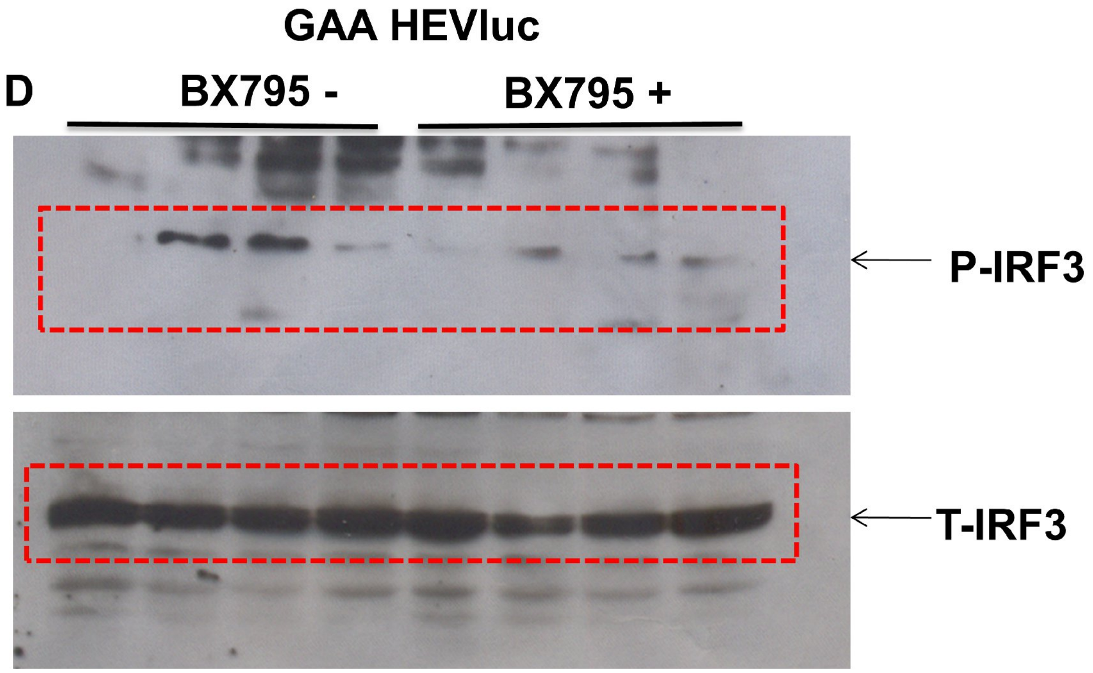

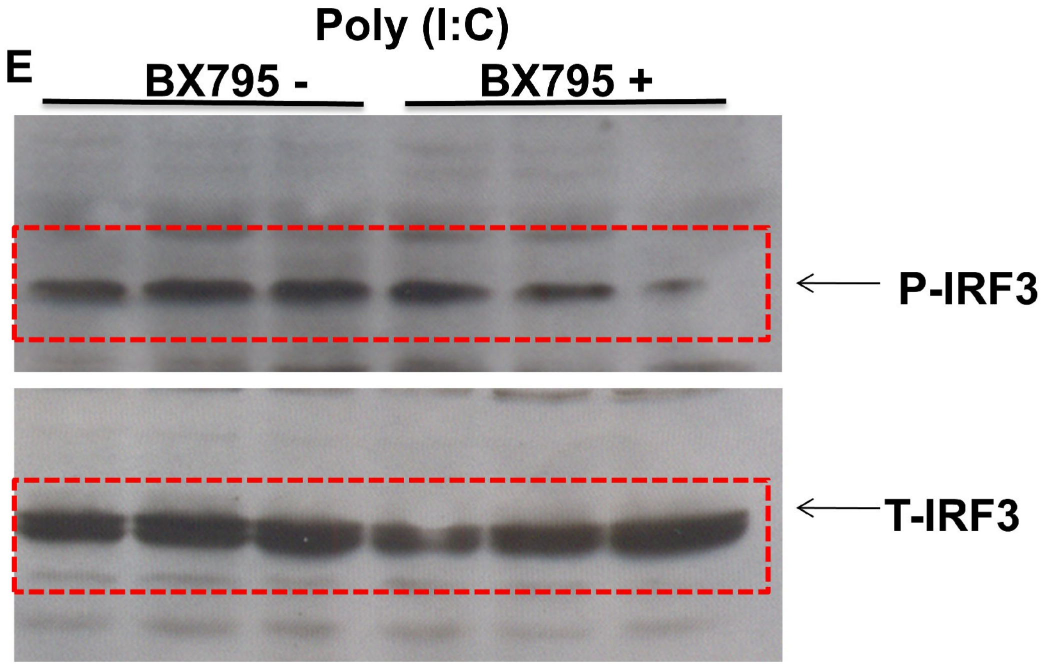

Supplementary figure 1. Full length blots of (A) RIG-I and corresponding actin, (B) TLR3 from Huh7.5 cell lysates and (C-E) phosphorylated IRF3 (P-IRF3, Ser396), the corresponding reprobed total IRF3 (T-IRF3) from HepG2/C3A cells transfected with HEVluc RNA, GAA mutant RNA and poly I:C either in presence or absence of BX795 treatment.

Supplementary Table

Table S1. Primer sequences used for real-time PCR assays.

Primer	Orientation	Sequence	Product size (bp)
IFN-α	Sense	GCCTCGCCCTTTGCTTTACT	88
	Antisense	CTGTGGGTCTCAGGGAGATCA	
IFN-β	Sense	ATGACCAACAAGTGTCTCCTCC	68
	Antisense	GCTCATGGAAAGAGCTGTAGTG	
GAPDH	Sense	GTGGACCTGACCTGCCGTCT	153
	Antisense	GGAGGAGTGGGTGTCGCTGT	
ISG56	Sense	TCTCAGAGGAGCCTGGCTAAG	450
	Antisense	CCACACTGTATTTGGTGTCTAGG	
PKR	Sense	CAGGCACGACAAGCATAGAA	406
	Antisense	CTACTCCCTGCTTCTGACGG	
Mx1	Sense	ATGATTGTCAAGTGCCG	127
	Antisense	GCCTTTCCTTCCTCCA	
Mda5	Sense	CAGAAGGAAGTGTCAGCTGCTTAG	106
	Antisense	TGCTGCCACATTCTCTTCATCT	
